# EM Dipeptide Enhances Milk Protein Secretion: Evidence from Integrated Metabolomic and Transcriptomic Analysis

**DOI:** 10.3390/metabo15070476

**Published:** 2025-07-14

**Authors:** Yuqing Liu, Yuhao Yan, Runjun Yang, Xiaohui Li, Chuang Zhai, Xuan Wu, Xibi Fang, Boqun Liu

**Affiliations:** 1 College of Food Science and Engineering, Jilin University, Changchun 130062, China; yql23@mails.jlu.edu.cn (Y.L.);; 2 College of Animal Science, Jilin University, Changchun 130062, China; 3 Center of Animal Experiment, College of Basic Medical Sciences, Jilin University, Changchun 130021, China

**Keywords:** methionine-containing dipeptide (EM), mammary epithelial cells, milk protein synthesis, transcriptomics, metabolomics, nutrient transport, lactogenic activity

## Abstract

**Background/Objectives:** Breast milk provides essential nutrition and immune protection to support infant growth and development. However, insufficient breast milk remains a serious issue, and bioactive peptides represent a potential strategy to promote lactation. In this study, we investigated the impact of a methionine-containing dipeptide, EM, on MCF-10A mammary epithelial cells. **Methods:** MCF-10A cells were treated with EM, and cell proliferation and the expression of key milk protein genes were assessed. Integrated transcriptomic and untargeted metabolomic analyses were performed to identify EM-induced changes in metabolic and gene expression pathways. **Results:** EM treatment significantly enhanced cell proliferation and upregulated the expression of key milk protein genes (CSN1S1 (casein alpha-S1, encoding alpha-S1 casein), CSN2 (casein beta, encoding beta-casein), and CSN3 (casein kappa, encoding kappa-casein)) at both transcriptional and protein levels compared to controls. Integrated transcriptomic and metabolomic analyses revealed that EM reprogrammed amino acid metabolism, lipid biosynthesis, and nutrient transport pathways. Core genes such as SLC7A11, APOE, and ABCA1 were identified as critical nodes linking metabolic and transcriptional networks. **Conclusions:** These findings indicate that EM may promote lactogenic activity by modulating metabolic and transcriptional networks *in vitro*, highlighting the potential of dipeptide-based nutritional interventions, which warrants further *in vivo* validation.

## 1. Introduction

Breast milk is the optimal source of nutrition and immune protection for newborns, providing essential macronutrients, bioactive molecules, and immunoglobulins, critical for infant growth and development [1,2,3]. The protein content of breast milk, particularly caseins and whey proteins, plays a pivotal role in supporting neonatal health, influencing gastrointestinal development, immune system maturation, and neurodevelopment [4,5]. Milk protein synthesis is tightly regulated by the uptake and metabolism of amino acids in mammary epithelial cells (MECs), which in turn activate key intracellular signaling pathways, notably the mammalian target of rapamycin (mTOR) and signal transducer and activator of transcription 5 (STAT5) pathways [6,7,8,9,10]. These pathways orchestrate the transcription of milk protein genes and the translation of caseins and whey proteins in response to nutrient and hormonal cues [11,12]. Although the exact prevalence is uncertain, challenges related to insufficient lactation are frequently reported in both clinical and community settings, particularly during early postpartum [13].

Recent studies in ruminant species have demonstrated that small bioactive peptides can enhance lactation performance by stimulating these key pathways [7,14,15]. Specifically, methionine-containing dipeptides, such as Met-Lys, Met-Phe, and Met-Trp, have been shown to promote casein synthesis in bovine mammary epithelial cells by activating nutrient-sensing and anabolic signaling cascades [7,16,17]. These findings suggest that methionine-rich dipeptides may serve as promising functional ingredients for improving milk production.

However, despite compelling evidence from livestock models, the effects of methionine-containing dipeptides on human mammary epithelial cells (MCF-10A) remain poorly understood [18,19]. Whether similar mechanisms of enhanced amino acid transport, metabolic reprogramming, and milk protein synthesis occur in human cells has not been systematically investigated [20]. Given the differences between ruminant and human lactation physiology, direct evaluation in human cell models is necessary to assess the potential applicability of dipeptide supplementation strategies [21,22].

In this context, we selected a methionine-containing dipeptide, EM, characterized by high transport efficiency via the peptide transporter 1 (PepT1) peptide transporter, to explore its lactogenic effects in MCF-10A [23,24]. We hypothesized that EM could enhance cell proliferation and stimulate milk protein synthesis by reprogramming transcriptional and metabolic pathways [25]. To test this hypothesis, we conducted an integrated omics study combining transcriptomic and untargeted metabolomic analyses to elucidate the molecular mechanisms underlying EM-induced lactogenic activity. The findings aim to provide new insights into the functional roles of dipeptides in human lactation and offer a basis for developing novel nutritional interventions for maternal and infant health.

## 2. Materials and Methods

### 2.1. Cell Culture and Treatment

The MCF-10A normal human mammary epithelial cell line (non-tumorigenic) was purchased from the Cell Resource Center, IBMS, CAMS/PUMC (Beijing, China). The MCF-10A cells were cultured in Dulbecco’s Modified Eagle Medium/Nutrient Mixture F-12 (DMEM/F-12; Corning, Corning, NY, USA) supplemented with 10 μg/mL insulin (Solarbio, Beijing, China), 0.5 μg/mL hydrocortisone (Solarbio, Beijing, China), 20 ng/mL epidermal growth factor (EGF; Peprotech, Rocky Hill, NJ, USA), 100 ng/mL cholera toxin (Absin, Shanghai, China), and 10% fetal bovine serum (FBS; Tianhang Biotechnology, Huzhou, Zhejiang, China) under the condition of 37 °C and 5% CO_2_. The MCF-10A cells were subcultured using 0.05% trypsin (Thermo Fisher Scientific, Waltham, MA, USA) upon reaching 80–90% confluence. To detect the effect of methionine on the MCF-10A cells, they were cultured in six-well plates (Biofil, Guangzhou, Guangdong, China). When the cells converged to 70%, the culture medium was replaced with serum-free DMEM/F-12 for 12 h to synchronize the cell cycle and simulate a nutrient-deprived environment [26]. To simulate lactation conditions, cells were treated with human prolactin (hPRL, Y-S Biotechnology, Shanghai, China) and various concentrations of EM (0, 50, 100, and 150 μg/mL) for 24 h to induce milk protein expression [27].

### 2.2. Cell Viability

To evaluate the effect of EM concentration on MCF-10A cell viability, cells were cultured in 96-well plates (Biofil, Guangzhou, Guangdong, China) and cultured until approximately 70% confluence. Serum starvation was then performed for 12 h to synchronize cell growth. Cells were subsequently treated with EM at concentrations of 0, 50, 100, and 150 μg/mL, together with prolactin (1 μg/mL) to simulate lactogenic conditions. Each treatment was performed in sextuplicate (*n* = 6). At 24 and 48 h post-treatment, the culture medium was removed, and cells were washed once with Phosphate-buffered saline (PBS; Thermo Fisher Scientific, Waltham, MA, USA). A total of 100 μL of Cell Counting Kit-8 (CCK-8) working solution (1:9 dilution of CCK-8 reagent (Beyotime, Shanghai, China) in complete medium) was added to each well. After 30 min of incubation at 37 °C, absorbance was measured at 450 nm using a microplate reader (YongChuang SM600, Shanghai, China) [26]. Cell viability was calculated based on absorbance values.

### 2.3. 5-Ethynyl-2′-Deoxyuridine (EdU) Incorporation Assay for Cell Proliferation

To evaluate the effect of EM dipeptide methionine concentration on MCF-10A cell proliferation, an EdU incorporation assay was conducted using a commercial kit (Beyotime, Shanghai, China). Cells were cultured in 24-well plates (Biofil, Guangzhou, Guangdong, China) and treated accordingly. After EdU labeling and nuclear counterstaining with Hoechst, fluorescence images were acquired under an inverted fluorescence microscope (Nikon TE2000, Tokyo, Japan) using identical exposure settings (exposure time: 500 ms; gain: 1.0) to ensure consistency across samples [28]. Fluorescence images were analyzed using ImageJ software (version 1.53c, NIH, Bethesda, MD, USA). Raw image files were first converted to 8-bit grayscale, and background was subtracted using the “Rolling Ball” algorithm (radius = 50 pixels). Regions of interest (ROIs) corresponding to individual cells or specific staining areas were selected either manually or automatically. Mean fluorescence intensity (MFI) was measured for each ROI using the “Measure” function.

For cell counting, Hoechst-stained nuclei were identified using automatic thresholding and counted with the “Analyze Particles” function (size: 50–1000 pixels^2^, circularity: 0.5–1.0). EdU-positive cells were similarly identified in the red fluorescence channel, and the percentage of EdU-positive cells was calculated by dividing the number of EdU-positive cells by the total number of nuclei.

### 2.4. RNA Extraction and Quantitative Real-Time PCR (qRT-PCR)

Total RNA was extracted from MCF-10A cells using a commercial kit (FastPure Cell/Tissue Total RNA Isolation Kit; Vazyme, Nanjing, Jiangsu, China), and RNA concentration was determined using a NanoDrop 2000 spectrophotometer (Thermo Fisher Scientific, Waltham, MA, USA). cDNA was synthesized from 1 μg of total RNA using a reverse transcription kit (Tiangen Biotech, Beijing, China). Quantitative real-time PCR (qRT-PCR) was performed using gene-specific primers designed with Primer Premier 6.0 (Premier Biosoft, Palo Alto, CA, USA) and SYBR Green Master Mix (ABclonal, Wuhan, Hubei, China) on a standard qRT-PCR platform. The cycling conditions were as follows: initial denaturation at 95 °C for 3 min, followed by 40 cycles of 95 °C for 5 s and 60 °C for 30 s. Relative gene expression levels were calculated using the 2^−ΔΔCt^ method [29]. The internal reference gene is *β-actin* (primer sequences shown in Table 1).

### 2.5. Immunofluorescence Assay

MCF-10A cells at appropriate density were seeded into glass-bottom culture dishes (Nest, Wuxi, Jiangsu, China). After treatment with EM, when cell density reached approximately 50%, cells were fixed with 4% paraformaldehyde (Biosharp, Hefei, Anhui, China) for 22 min, followed by permeabilization with 0.1% Triton X-100 (Bio Basic, Toronto, ON, Canada) for 5–10 min [30]. Cells were then blocked with 5% bovine serum albumin (BSA; Sigma-Aldrich, Darmstadt, Germany) for 30 min at 4 °C. The cells were incubated overnight at 4 °C with anti-beta-casein (CSN2) primary antibody (Proteintech, Wuhan, Hubei, China). After washing three times with PBST (PBS containing 0.1% Tween-20), cells were incubated with fluorescein isothiocyanate (FITC)-conjugated secondary antibody (Proteintech, Wuhan, Hubei, China) at 37 °C for 2 h in the dark. Nuclei were counterstained with Hoechst (Beyotime, Shanghai, China) for 10 min. Fluorescence signals were visualized using an inverted fluorescence microscope (Nikon TE2000, Tokyo, Japan).

### 2.6. RNA-Seq and Transcriptomic Data Analysis

MCF-10A cells were treated with 100 μg/mL of EM, and total RNA was extracted using TRIzol reagent (Invitrogen, Carlsbad, CA, USA) according to the manufacturer’s protocol [31]. Transcriptome sequencing and bioinformatic analysis were performed by Kaitai Biotechnology Co., Ltd. (Hangzhou, Zhejiang, China). Each group included three biological replicates (*n* = 6 in total). RNA libraries were prepared using the Illumina TruSeq RNA Sample Preparation Kit and sequenced on the Illumina platform. Raw sequencing reads underwent quality control and adapter trimming. Sequencing metrics, including raw and clean read counts, Q20, and Q30 values, are listed in Table 2. Clean reads were aligned to the human reference genome (GRCh38) using *HISAT2* (v2.2.1; Daehwan Kim Lab, Baltimore, MD, USA), and alignment statistics are provided in Table 3. Gene expression levels were quantified using *HTSeq* (v2.0.1; European Molecular Biology Laboratory, Heidelberg, Baden-Württemberg, Germany), and differential expression analysis was performed using the *edgeR* package (v3.42.4; Walter and Eliza Hall Institute of Medical Research, Melbourne, Victoria, Australia). Genes with an adjusted *p*-value (false discovery rate, FDR) < 0.05, calculated using the Benjamini–Hochberg procedure, were considered significantly differentially expressed. Functional enrichment analysis of Differentially expressed genes (DEGs) was carried out using the clusterProfiler *R* package (v4.2.1), based on the Gene Ontology (GO) (http://geneontology.org/, accessed on 7 July 2025) and Kyoto Encyclopedia of Genes and Genomes (KEGG) databases (https://www.genome.jp/kegg/, accessed on 7 July 2025), with a *p*-value cutoff of 0.05.

### 2.7. Untargeted Metabolomics Analysis

MCF-10A cells were treated with 100 μg/mL of EM, as described in Section 2.1. Untargeted metabolomic profiling was performed by Kaitai Biotechnology Co., Ltd. (Hangzhou, Zhejiang, China), with three biological replicates per group (*n* = 3 per group, 6 in total). Raw Liquid Chromatography-Tandem Mass Spectrometry (LC-MS/MS) data were preprocessed for statistical analysis. Principal Component Analysis (PCA) and Partial Least Squares-Discriminant Analysis (PLS-DA) were performed to assess metabolic variation, and differential metabolites were identified using *t*-tests or ANOVA (*p* < 0.05). Identified metabolites were annotated and analyzed using KEGG and MetaboAnalyst for pathway and network enrichment [32].

### 2.8. Multi-Omics Integration of Transcriptome and Metabolome

To elucidate the coordinated molecular mechanisms underlying the lactogenic effect of EM, transcriptomic and metabolomic datasets were integrated through a pathway- and correlation-based strategy. DEGs and significantly altered metabolites identified from transcriptome and untargeted metabolomics analyses, respectively, were first annotated using KEGG identifiers. Shared molecules were identified by mapping differentially expressed genes and altered metabolites to common KEGG pathways using compound–gene co-annotation in MetaboAnalyst 5.0. These matched features were used to identify shared or functionally related pathways. Joint enrichment analysis was performed using MetaboAnalyst 5.0, incorporating both transcript and metabolite data to visualize coordinated biological processes. Metabolites and DEGs involved in common pathways were subjected to correlation analysis using Pearson correlation coefficients, with |r| > 0.8 and *p* < 0.05 set as significance thresholds. Strongly correlated gene–metabolite pairs were used to construct interaction networks.

Network visualization and topology analysis were conducted using Cytoscape 3.9.1 [33]. Lactation-related pathways, including those involved in amino acid transport and metabolism, lipid biosynthesis, and protein synthesis, were particularly highlighted to identify functional modules potentially regulated by EM.

### 2.9. Quantitative Real-Time PCR (qRT-PCR) Validation of Selected DEGs

To validate the transcriptomic findings, we selected 50 DEGs associated with 17 lactation-related KEGG pathways (as shown in Table 4) for qRT-PCR analysis. MCF-10A cells were seeded in 6-well plates and cultured until reaching approximately 70% confluence. Subsequently, cells were serum-starved for 12 h, consistent with the conditions described in Section 2.1. Following starvation, cells were treated with one of the following conditions in the presence of prolactin to induce lactogenic signaling: 100 μg/mL EM, 100 μg/mL EM-D (an isomeric form of EM), 33.4 μg/mL EMEMEM (a trimeric form of EM). A control group treated with prolactin alone was included to account for the effects of lactogenic stimulation. qRT-PCR analysis of the 50 selected DEGs was performed as described in Section 2.4, using *β-actin* as the reference gene. Gene-specific primers are listed in Table 1.

### 2.10. Statistical Analysis

All experiments were repeated at least three times independently, and the data are presented as mean ± standard error (SEM). Statistical analysis was performed using R language (v4.2.1), GraphPad Prism 9, and SPSS 26.0 software, and the significance threshold was set at *p* < 0.05.

## 3. Results

### 3.1. EM Promotes MCF-10A Cell Proliferation

**Figure 1:**
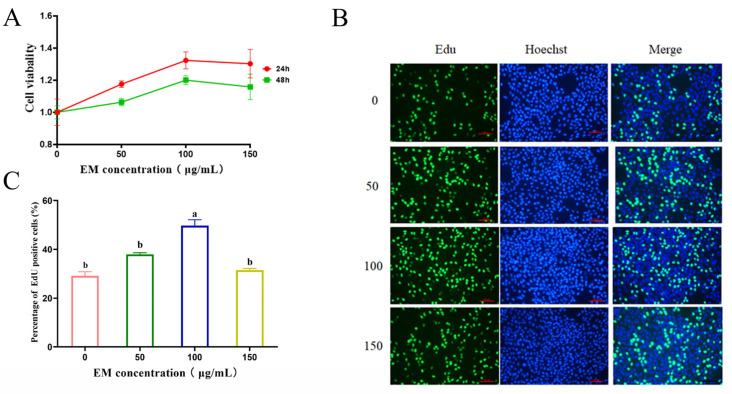
Effects of EM treatment on the proliferation of MCF-10A cells. MCF-10A cells were treated with varying concentrations of EM (0, 50, 100, and 150 μg/mL). (**A**) Cell viability was measured by CCK-8 assay at 24 and 48 h. No significant differences were observed between groups (*p* > 0.05). (**B**) EdU staining was performed to assess DNA synthesis-based cell proliferation after 24 h of treatment. Nuclei were stained with Hoechst (blue), and EdU-positive proliferating cells were labeled with green fluorescence. Scale bar = 100 μm. (**C**) Quantification of EdU-positive cells (%) under each treatment condition. Data are presented as mean ± SEM (*n* = 3). Statistical analysis was performed using one-way ANOVA followed by Tukey’s multiple comparisons test. Different lowercase letters indicate significant differences between groups (*p* < 0.05).

To determine whether EM promotes the proliferation of mammary epithelial cells—a critical step for subsequent milk protein synthesis—CCK-8 and EdU assays were performed. Cells were serum-starved for 12 h prior to treatment, and viability was evaluated accordingly. EM treatment enhanced MCF-10A cell proliferation in a dose-dependent manner, with the effect observed at 100 μg/mL after both 24 and 48 h. At 150 μg/mL, cell viability showed a slight decrease, possibly indicating cytotoxicity at higher concentrations (Figure 1A). EdU staining further supported these findings, with a marked increase in DNA-synthesizing cells at 100 μg/mL (Figure 1B), as confirmed by quantification in Figure 1C (*p* < 0.05). These results suggest that EM promotes mammary epithelial cell proliferation, which may contribute to greater epithelial cell numbers, improved mammary gland structure and function, and ultimately, enhanced milk protein synthesis [28,34].

### 3.2. EM Enhances Milk Protein Synthesis at the Transcriptional and Protein Levels

**Figure 2:**
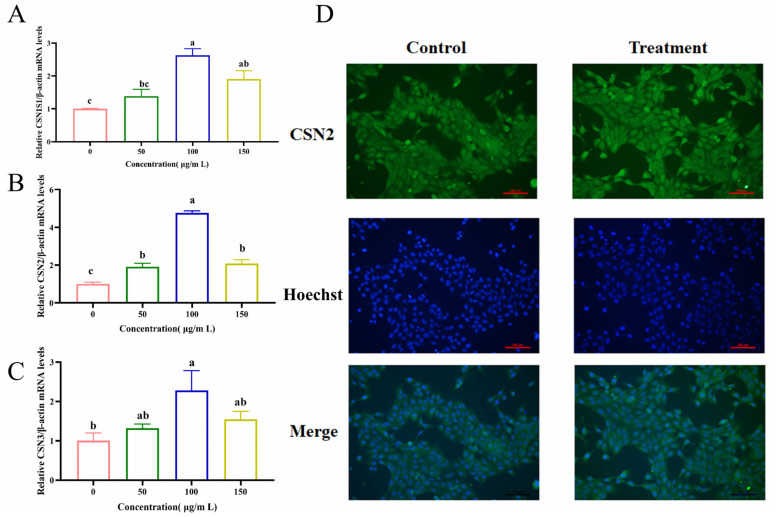
EM treatment promotes the expression of milk protein genes and β-casein in MCF-10A cells. (**A**–**C**) qRT-PCR analysis of the mRNA expression levels of CSN1S1 (**A**), CSN2 (**B**), and CSN3 (**C**) in MCF-10A cells treated with different concentrations of EM (0, 50, 100, and 150 μg/mL) for 24 h. Gene expression was normalized to *β-actin* and is presented as fold change relative to the untreated control group. Data are shown as mean ± SEM (*n* = 3). One-way ANOVA followed by Tukey’s post hoc test was used to determine statistical significance. Bars with different lowercase letters indicate significant differences (*p* < 0.05). (**D**) Immunofluorescence staining of β-casein (CSN2) protein in MCF-10A cells after treatment with 100 μg/mL EM or vehicle control. β-casein was labeled with FITC-conjugated secondary antibody (green), and nuclei were counterstained with Hoechst (blue). Merged images show clear cytoplasmic CSN2 signal. Images were captured using an inverted fluorescence microscope under identical exposure settings. Scale bar = 100 μm.

To determine whether EM promotes lactation-related functional protein synthesis, we measured the mRNA expression of three major milk protein genes—CSN1S1, CSN2, and CSN3—using qRT- PCR. As shown in Figure 2A–C, EM treatment significantly upregulated the expression of all three genes in a dose-dependent manner. The highest induction of CSN1S1 (*p* < 0.05), CSN2 (*p* < 0.05), and CSN3 (*p* > 0.05) was observed at 100 μg/mL. A slight reduction at 150 μg/mL was observed, though expression remained significantly elevated compared to the untreated control (Figure 2A–C).

To validate these transcriptional changes at the protein level, we performed immunofluorescence staining for β-casein (CSN2). EM-treated cells exhibited visibly enhanced green fluorescence compared to controls, indicating increased β-casein expression (Figure 2D). Importantly, all imaging was performed under identical exposure settings, and cells were cultured to a controlled confluency (50%) prior to fixation to ensure consistent cell density across groups. Nuclei were counterstained with Hoechst to assess cell number, which confirmed comparable nuclear density between treatment and control groups. These findings confirm that the increase in β-casein signal reflects elevated per-cell protein expression, rather than a change in cell number. Thus, EM enhances milk protein synthesis at both the transcriptional and translational levels in MCF-10A cells.

### 3.3. EM Reprograms Transcription to Promote Lactation

**Figure 3:**
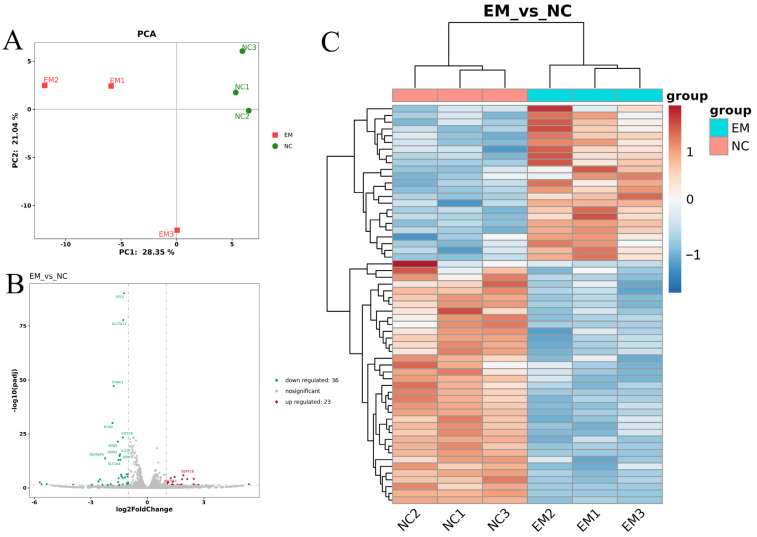
Transcriptome data quality control and sample distribution analysis. (**A**) Principal component analysis (PCA) plot demonstrating clear separation between EM-treated and control (NC) groups. (**B**) Volcano plot showing DEGs between groups, with significantly upregulated (red) and downregulated (green) genes (adjusted *p* < 0.05, |log_2_FC| ≥ 1). (**C**) Hierarchical clustering heatmap of differentially expressed genes, showing distinct clustering of EM and NC samples based on gene expression profiles.

To investigate the global transcriptomic changes induced by EM treatment, we performed RNA sequencing analysis on MCF-10A cells treated with EM and untreated controls (NC). Principal component analysis (PCA) revealed a clear separation between the EM and NC groups, with PC1 and PC2 explaining 28.35% and 21.04% of the variance, respectively (Figure 3A), indicating distinct overall gene expression profiles.

Differential expression analysis identified a total of 59 DEGs between the two groups, including 23 upregulated and 36 downregulated genes (adjusted *p* < 0.05, |log_2_FC| ≥ 1) (Figure 3B). Notably, several DEGs such as CSN2, CSN1S1, and GAMT were significantly upregulated in the EM group, suggesting potential roles in milk protein synthesis and metabolic regulation.

Hierarchical clustering of the DEGs demonstrated clear within-group consistency and distinct separation between EM-treated and control samples based on their expression profiles (Figure 3C). The heatmap further illustrates that the transcriptional response to EM treatment involved coordinated regulation of genes associated with epithelial function and biosynthetic activity.

**Figure 4:**
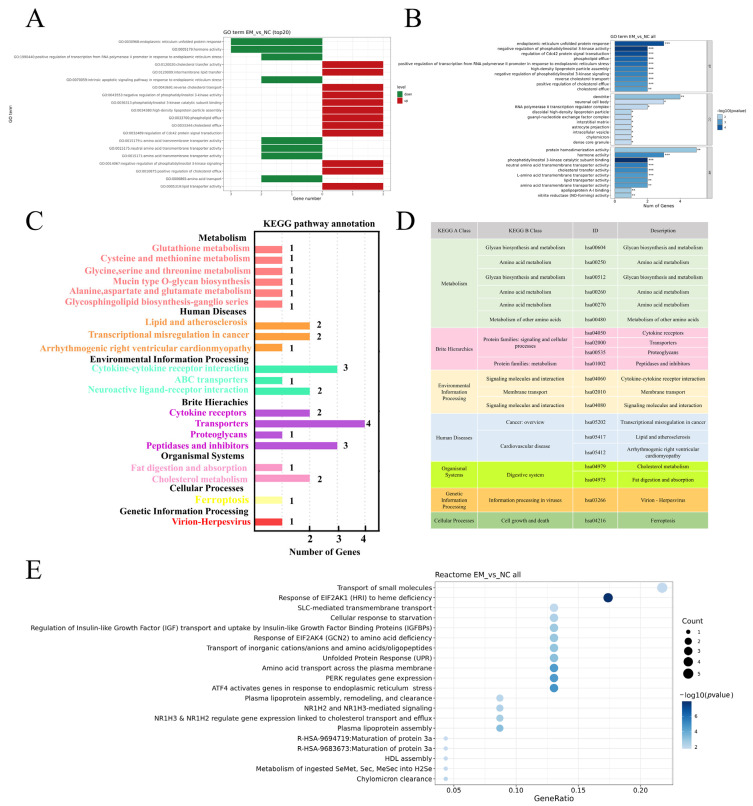
Transcriptome analysis of EM-treated MCF-10A cells versus control. (**A**) GO enrichment of the top 20 (DEGs), showing biological processes enriched for upregulated (red) and downregulated (green) genes. (**B**) GO enrichment categorized by biological process (BP), cellular component (CC), and molecular function (MF); bar length indicates gene count, and color reflects −log_10_ (*p*-value). (**C**) KEGG pathway enrichment grouped by major biological functions. (**D**) KEGG classification summary showing pathway IDs, categories, and functional descriptions. (**E**) Reactome enrichment dot plot; dot size denotes gene count and color indicates enrichment significance. * *p* < 0.05, ** *p* < 0.01, *** *p* < 0.001.

To explore the biological significance of the (DEGs) between EM-treated and control (NC) groups, we conducted GO, KEGG, and Reactome enrichment analyses. GO enrichment analysis showed that upregulated genes were enriched in biological processes such as endoplasmic reticulum unfolded protein response, amino acid transmembrane transport, and transcriptional regulation in response to stress. Downregulated genes were associated with lipid transport and cholesterol efflux (Figure 4A,B). KEGG analysis identified enriched pathways including amino acid metabolism, ABC transporters, cytokine–cytokine receptor interaction, and protein digestion and absorption. These pathways were mainly classified under metabolism, environmental information processing, and organismal systems (Figure 4C,D). Reactome analysis revealed significant enrichment in pathways such as transport of small molecules, EIF2AK1/EIF2AK4 response to stress, ATF4 and PERK signaling, SLC-mediated transmembrane transport, IGF transport and uptake, and plasma lipoprotein assembly (Figure 4E).

### 3.4. EM Reprograms Metabolism to Support Milk Component Synthesis

**Figure 5:**
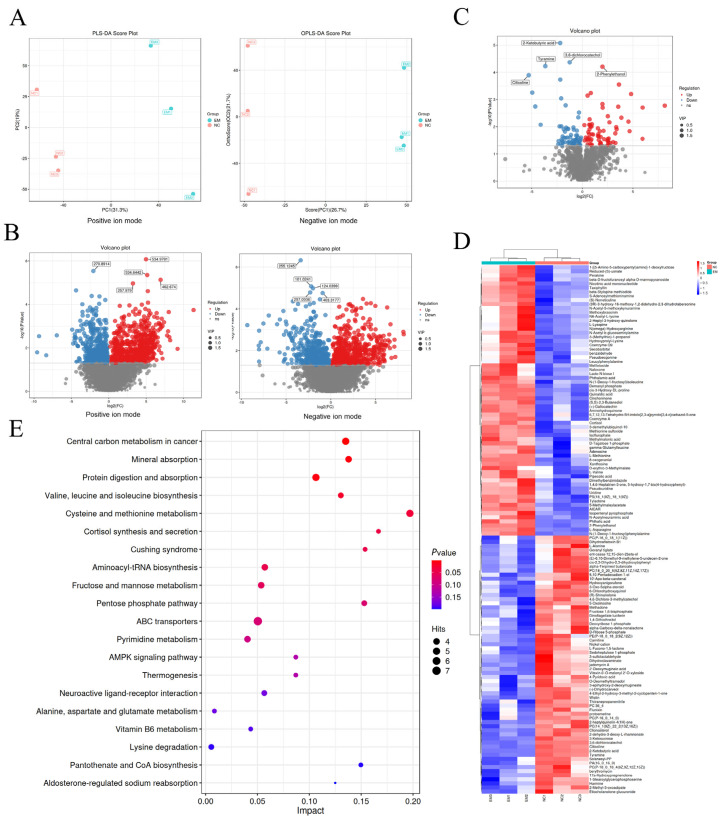
Metabolomic profiling of EM-treated mammary epithelial cells. (**A**) PLS-DA score plots based on LC-MS/MS data in both positive and negative ion modes, illustrating sample distribution between EM and NC groups. (**B**) Volcano plots of significantly altered metabolites (VIP > 1, |log_2_FC| > 1, *p* < 0.05) under positive and negative ionization. (**C**) Combined volcano plot showing the overall distribution of significantly changed metabolites across both ionization modes. (**D**) Heatmap of all differential metabolites clustered by treatment group. Each row represents a metabolite and each column a sample; color scale indicates z-score normalized intensity. (**E**) KEGG pathway enrichment bubble plot of differential metabolites. Dot size represents the number of hits per pathway, and color reflects statistical significance (*p*-value).

To investigate the metabolic effects of EM treatment, untargeted metabolomic profiling was performed using LC-MS/MS in both positive and negative ion modes. As shown in Figure 5A, PLS-DA score plots demonstrated clear separation between the EM and NC groups, indicating distinct global metabolic profiles. Differential metabolite screening is shown in Figure 5B, with volcano plots illustrating numerous significantly altered metabolites in both ionization modes (VIP > 1, |log_2_FC| > 1, *p* < 0.05). To identify common metabolic alterations, a combined volcano plot (Figure 5C) was generated, showing the total number and distribution of significantly upregulated and downregulated metabolites across modes. Unsupervised hierarchical clustering of all differential metabolites is shown in Figure 5D, where metabolite abundance patterns clearly distinguished EM from NC samples, indicating consistent treatment-dependent metabolic changes. Figure 5E presents the KEGG pathway enrichment analysis. EM-induced differential metabolites were significantly enriched in pathways related to amino acid metabolism (e.g., valine, leucine, isoleucine biosynthesis, cysteine and methionine metabolism), protein and mineral absorption, ABC transporters, and AMPK signaling. A detailed summary of the enriched KEGG pathways and associated differential metabolites is provided in Table 5.

### 3.5. Integrated Omics Reveal Core Lactogenic Regulatory Networks Induced by EM

**Figure 6:**
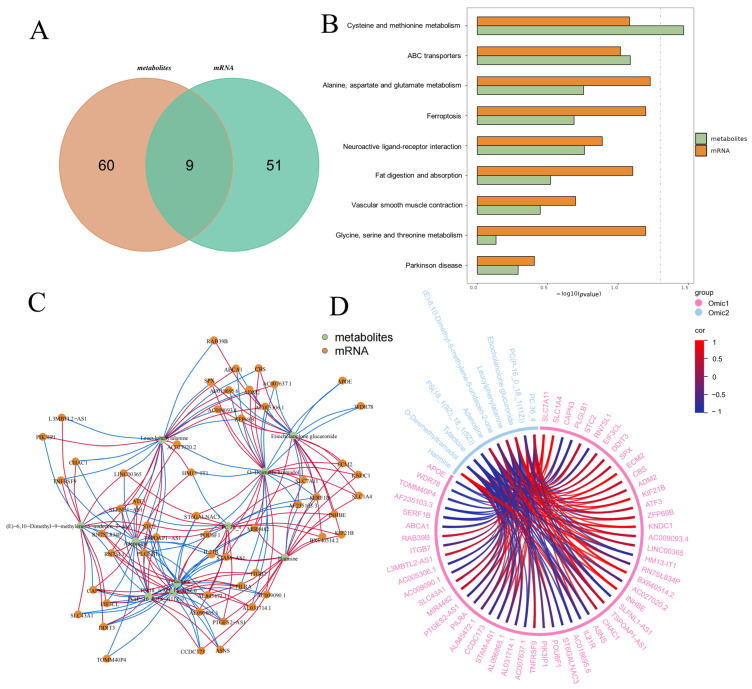
Multi-omics integrative analysis reveals key transcript-metabolite interactions induced by EM treatment. (**A**) Venn diagram showing the overlap of significantly enriched KEGG pathways identified from transcriptomic and metabolomic datasets. (**B**) KEGG pathway enrichment analysis of differentially expressed genes and metabolites. Orange bars represent gene-enriched pathways, and green bars represent metabolite-enriched pathways. (**C**) Multi-omics network diagram showing the correlations between differential genes and metabolites. Nodes represent genes (orange) and metabolites (green); edges indicate significant correlations, with red lines for positive and blue lines for negative associations. (**D**) Chord diagram displaying significantly correlated mRNA-metabolite pairs shared across omics datasets. The thickness and color of connecting lines represent correlation strength and direction (red: positive; blue: negative).

To elucidate the molecular mechanisms underlying EM-induced lactogenic and metabolic changes in mammary epithelial cells, we performed integrative analyses of transcriptomic and metabolomic datasets. A Venn diagram (Figure 6A) revealed that 9 KEGG pathways were significantly altered in both omics layers, suggesting the presence of shared regulatory targets influenced by EM. KEGG pathway enrichment analysis (Figure 6B) demonstrated that several metabolic pathways—including cysteine and methionine metabolism, ABC transporters, glutamate and alanine metabolism, and fat digestion and absorption—were enriched in both mRNA and metabolite datasets. This overlap indicates a potential convergence of transcriptional and metabolic regulation in amino acid and lipid handling under EM treatment. To further investigate transcript–metabolite relationships, we constructed a multi-omics correlation network (Figure 6C). Notably, multiple metabolites were found to be significantly correlated with key genes involved in cellular metabolism, transport, and signaling. Red and blue edges in the network represent positive and negative correlations, respectively, highlighting the complex and bidirectional nature of EM-driven regulatory interactions.

Moreover, a chord diagram (Figure 6D) illustrated robust associations between selected mRNAs and metabolites across both omics layers. Key lactogenic and metabolic regulators (e.g., CSN2, SLC family genes, ABC transporters) were tightly linked to small-molecule metabolites involved in energy metabolism, implying a coordinated regulatory mechanism whereby EM modulates both gene expression and metabolic outputs to support mammary cell function. Thus, even with a limited number of directly overlapping features, the consistent enrichment of shared pathways and strong cross-omic correlations provide robust evidence for a coordinated lactogenic regulatory network. These findings collectively underscore the systemic impact of EM on cellular metabolic pathways and gene networks, offering insight into the molecular underpinnings of its lactogenic and metabolic effects.

### 3.6. Validation of Differential Genes of Differential Metabolites

**Figure 7:**
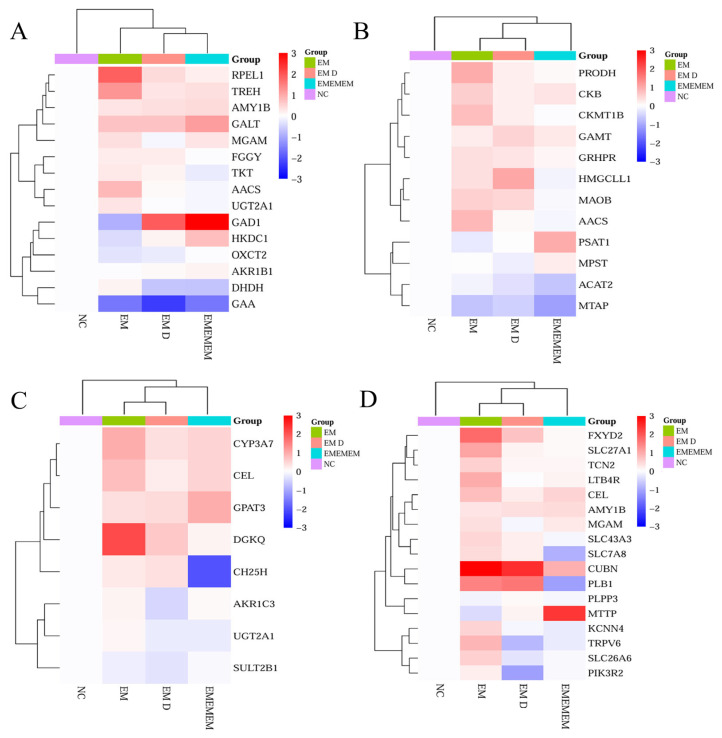
Heatmap of qRT-PCR validation results for differentially expressed genes. (**A**) Carbohydrate metabolism. (**B**) Amino acid metabolism. (**C**) Lipid metabolism. (**D**) Nutrient absorption.

To validate the transcriptomic findings, representative DEGs involved in carbohydrate, amino acid, lipid metabolism, and nutrient absorption were evaluated by qRT-PCR across four groups (NC, EM, EM D-configuration, and EMEMEM). Gene expression levels are presented as normalized log_2_(fold change) and visualized using cluster-based heatmaps. In the carbohydrate metabolism module, 15 genes were analyzed, including those associated with sugar interconversion (GALT, FGGY), starch and sucrose hydrolysis (AMY1B, TREH, MGAM), the pentose phosphate pathway (TKT, RPEL1), and energy-related enzymes (HKDC1, GAD1, OXCT2) (Figure 7A). For amino acid metabolism, 12 genes were examined, encompassing pathways related to arginine and proline metabolism (PRODH, CKB, CKMT1B), methionine metabolism (GAMT), glycine and serine utilization (GRHPR, PSAT1, MAOB), and other auxiliary processes (AACS, MPST, MTAP) (Figure 7B). Eight genes involved in lipid metabolism were tested, including those related to bile acid biosynthesis (CH25H), lipid hydrolysis (CEL), glycerolipid remodeling (DGKQ), and steroid hormone metabolism (CYP3A7, AKR1C3, SULT2B1) (Figure 7C). For nutrient absorption, 17 genes were assessed, including those involved in mineral uptake (TRPV6, SLC26A6), vitamin and lipid absorption (PLB1, PLPP3, MTTP, CUBN), amino acid transport (SLC7A8, KCNN4), and carbohydrate digestion (AMY1B, MGAM). Additional regulators (PIK3R2, FXYD2) were included based on their KEGG pathway relevance (Figure 7D).

The qRT-PCR validation of key transport genes revealed that the promotion of lactation by EM is governed by a complex regulatory network rather than a single signaling pathway. The coordinated upregulation of genes involved in carbohydrate metabolism, amino acid utilization, lipid remodeling, and nutrient absorption underscores the multifactorial nature of lactogenic regulation. These findings highlight lactation as a highly integrated physiological process requiring synchronized metabolic and transcriptional support.

## 4. Discussion

In this study, we demonstrated that the methionine-containing dipeptide EM significantly enhanced the proliferation ofMCF-10A and promoted the synthesis of major milk proteins at both the transcriptional and protein levels [7,25]. Integrated transcriptomic and metabolomic analyses further revealed that EM treatment induced a coordinated reprogramming of metabolic and gene expression pathways associated with amino acid metabolism, nutrient absorption, and biosynthetic processes essential for lactation [35].

Our findings are consistent with previous reports that small peptides, particularly methionine-containing dipeptides, can activate key signaling pathways such as mTOR and PI3K-AKT to stimulate milk protein synthesis in ruminants (e.g., bovine and caprine mammary gland cells) [25]. However, most existing studies have been confined to animal models. Here, to our knowledge, this study provides the first demonstration of EM’s lactogenic effects in an *in vitro* human mammary epithelial model, although we acknowledge that despite a comprehensive literature search, some relevant reports may not have been captured. This highlights the potential of dipeptide-based strategies to modulate human milk biosynthesis at the cellular level [36].

Through RNA-seq analysis, we observed that EM treatment significantly upregulated genes involved in carbohydrate, amino acid, and lipid metabolism, as well as nutrient transport pathways such as ABC transporters [36,37,38]. These changes are critical for supporting the elevated energy and substrate demands of lactating cells. Metabolomic profiling further confirmed broad remodeling of amino acid pools, lipid intermediates, and carbohydrate derivatives, consistent with enhanced biosynthetic capacity. Notably, cysteine and methionine metabolism, glycine, serine, and threonine metabolism, and alanine, aspartate, and glutamate metabolism were among the most significantly affected pathways, underscoring the centrality of amino acid metabolism in EM-induced lactogenesis [39].

The integrated omics correlation analysis revealed strong associations between amino acid-derived metabolites and nutrient transporter genes, suggesting a synchronized regulation between metabolite availability and transporter expression [17,40,41]. Such coordinated responses are essential for efficient substrate uptake and utilization during milk component synthesis [42]. These findings imply that EM not only serves as a nutritional precursor but may also act as a metabolic signaling molecule to reprogram cellular metabolic fluxes toward lactation [14,43].

Importantly, our study addresses a critical knowledge gap regarding the impact of dipeptides on human mammary function [44,45]. Previous work has largely focused on free amino acid supplementation, overlooking the potential advantages of dipeptide forms, which exhibit higher bioavailability and faster cellular uptake via PepT1 transporters [46,47]. Our results suggest that dipeptide-based interventions may warrant investigation as potential interventions pending safety and efficacy studies, particularly in populations with increased nutritional needs [14].

Although MCF-10A is a widely used, non-tumorigenic human mammary epithelial line, it exhibits basal-like traits and lacks the specialized phenotype, hormonal responsiveness, and high secretory capacity of fully differentiated lactating cells [48,49]. Therefore, our *in vitro* findings must be interpreted with caution, and validation in primary human cells or *in vivo* lactation models will be essential. We employed three biological replicates per group for both transcriptomic and metabolomic analyses, in line with common exploratory omics practice; while clustering and quality control metrics support the robustness of major trends, this small sample size limits statistical power and generalizability [50]. EM concentrations (50, 100, 150 μg/mL) were chosen based on precedent in peptide bioactivity assays and our own CCK-8/EdU tests, which showed no cytotoxicity up to 150 μg/mL and optimal proliferative responses at 50–100 μg/mL. However, the systemic exposure, bioavailability, and safety of these doses remain untested *in vivo*, necessitating comprehensive pharmacokinetic and toxicity studies to establish physiological relevance and guide translational development. Secondly, while we identified key metabolic and transcriptional shifts, the upstream signaling cascades (e.g., mTORC1 activation, STAT5 phosphorylation) were not directly examined and warrant further investigation [14,25]. Finally, the long-term safety and efficacy of EM supplementation for promoting lactation remain to be explored.

In conclusion, this study provides new insights into the molecular mechanisms by which the methionine-containing dipeptide EM enhances lactogenic activity in human mammary epithelial cells. These findings suggest that EM holds promise as a functional ingredient for supporting lactation and maternal health. Future research should focus on elucidating the signaling pathways involved, validating the effects *in vivo*, and exploring its application in functional foods or nutritional supplements designed for breastfeeding mothers [38].

## 5. Conclusions

In summary, this study suggests that the methionine-containing dipeptide EM significantly promotes the proliferation of human mammary epithelial cells and enhances milk protein synthesis. Through integrated transcriptomic and metabolomic analyses, we revealed that EM induces metabolic and transcriptional reprogramming in pathways potentially relevant to amino acid metabolism, lipid biosynthesis, and nutrient transport, which may be associated with lactogenic functions. These findings provide new insights into the potential application of dipeptide-based strategies to improve lactation performance. Future research should focus on validating these effects *in vivo* and exploring the underlying signaling mechanisms to facilitate the development of functional foods for maternal and infant health.

## Figures and Tables

**Table 1:** The primer sequences used for qRT-PCR.

Gene Name	Primer Sequence (5′-3′)
*CSN1S1-F*	AGGGCACCTAATCAGAGGGT
*CSN1S1-R*	AATTGATGGCACTTACAGAACTGG
*CSN2-F*	GGAGAGGATGAACACCAGGATA
*CSN2-R*	GCTGAGCAAGAGGCAGAATG
*CSN3-F*	GCAGGTCCCTCAGCCTATTC
*CSN3-R*	ACAGCTCTCTGAGGGTAGGG
*β-actin-F*	AGACCTGTACGCCAACACAG
*β-actin-R*	CGCTCAGGAGGAGCAATGAT
*MPST-F*	GCCGTGTCACTGCTTGATG
*MPST-R*	GGTTCTCCTTGATGTCCTCGTA
*PSAT1-F*	GTGTGCTGACTATGTGGTGAC
*PSAT1-R*	GTTGAGGTTCCAGGTGCTTG
*MTAP-F*	TCAACTACCAGGCGAACATCT
*MTAP-R*	GGCACAAGAATGACTTCCATCA
*GAMT-F*	GCCCATTGATGAGCATTGGAT
*GAMT-R*	GGCGTGGTTCTTGATGAAGTT
*GRHPR-F*	CCAGATGTCCTGACAGATACCA
*GRHPR-R*	AGCCACCATTCTTCACTTCCT
*MAOB-F*	GGCAGGACTTACACTCTTAGGA
*MAOB-R*	AGACGCTCAACCTCATTCACT
*ACAT2-F*	GGCTCCTCACTTGGCTTACT
*ACAT2-R*	GCAACCTTGTCCTGATCTTCTC
*HMGCLL1-F*	GGTATGTGTCTTGTGCTCTGG
*HMGCLL1-R*	GCTAAGGCTTGTCCGTATGTG
*AACS-F*	TGGCTGTCAGTGCTGGAAG
*AACS-R*	CAGGAGGATGCTGCTCTTGA
*PRODH-F*	AGAGCACAAGGAGATGGAGTC
*PRODH-R*	CTCATTGGCGTAGAAGTAGGTG
*CKMT1B-F*	GTGGCTGGAGATGAGGAGAC
*CKMT1B-R*	TAGATCCGTGGTGTGCTTCAT
*CKB-F*	CTCATCGACGACCACTTCCT
*CKB-R*	ATTGTCATTGTGCCAGATACCG
*GAD1-F*	ACAGCCTGGAAGAGAAGAGTC
*GAD1-R*	TTGCGGACATAGTTGAGGAGTA
*OXCT2-F*	GTTCAACGGCGACCACTTC
*OXCT2-R*	CCACCTCCACGATCTCTTCC
*RPEL1-F*	TGCCATCAAACCAGGAACCT
*RPEL1-R*	TGTCAGAACCTACTCCACCATC
*GAA-F*	CGTTCATGCCGCCATACTG
*GAA-R*	TGTAGTCCAGGTCGTTCCAC
*AKR1B1-F*	GATCGCAGCCAAGCACAATA
*AKR1B1-R*	CAGCTCAACAAGGCACAGAC
*GALT-F*	GCGTGATGATCTAGCCTCCA
*GALT-R*	TCTGAGCCTGAGCAAGCATT
*UGT2A1-F*	CCATAGACCAACTCCTCTCACA
*UGT2A1-R*	GAGCAACAAGATCACCACAGAT
*TKT-F*	AGTGATGGCGTTGCTACAGA
*TKT-R*	GACCTGGAAGTCCTCATTGTTG
*DHDH-F*	TCGGGCAGAATTTGGGAAGA
*DHDH-R*	TGGAGGTGAACTGGACACAG
*FGGY-F*	GCTGGCTGTCATCTGTGGA
*FGGY-R*	ACTCTGGCATCTGGCTGTG
*AMY1B-F*	CGGCACAGTTATTCGCAAGT
*AMY1B-R*	GTCCTCGTTGATTGTCATGGTT
*MGAM-F*	TGACATACCGCACCACAGG
*MGAM-R*	GCAGCCACCATCTCATCATAC
*TREH-F*	TGCTACTTGACTCACACCAATG
*TREH-R*	AGCGATTCAGGAGGTAGTTCTT
*HKDC1-F*	GATGTGGTGAGCCGTCTGA
*HKDC1-R*	TGCCAGTTCCGATGATGACA
*CH25H-F*	GCTACAAGATCCACCCTGACT
*CH25H-R*	TGCCACACGAAGAACTCCAT
*CYP3A7-F*	TGCTCTAGTCAGAGTCCTTCAG
*CYP3A7-R*	GGCTCCACTTACGGTCTCAT
*AKR1C3-F*	GATGGTCACTTCATGCCTGTC
*AKR1C3-R*	CAGTCCAACCTGCTCCTCAT
*SULT2B1-F*	TCGGATGAAGGGCAAAGACA
*SULT2B1-R*	AGCAGCGTGTAGTTGGACAT
*GPAT3-F*	CTGGTTCTCGGCTTCATCCT
*GPAT3-R*	CTCAAAGTCCCTTCCTCGTAGA
*DGKQ-F*	CGTTCTCCGTACTGCTGTCT
*DGKQ-R*	GTCTGCCGTGTCGTTCTCT
*CEL-F*	CACATCTTCGCCAGCATCG
*CEL-R*	AGTCCACCACAGTCTTCTTCTT
*TRPV6-F*	GCCGAGATGAGCAGAACCT
*TRPV6-R*	CAGCCTCCATCAGCACCAT
*FXYD2-F*	GTGGACCCGTTCTACTATGACT
*FXYD2-R*	ATCTTCTGCTGAGGAGGATGAG
*SLC43A3-F*	CTGGTGTGGCTGTCTGTGA
*SLC43A3-R*	TGCTTCCTTCTGGTACTTCTGT
*SLC26A6-F*	ATCTTGCTGAACCTGGACCTT
*SLC26A6-R*	CCACACCACACCTCTGCTT
*PLB1-F*	TTGTGGATTCAGGCTCAAGAAC
*PLB1-R*	GAGAGGAGAACCGTGCTAAGT
*TCN2-F*	CTCTACCTGCTCGCTCTCAG
*TCN2-R*	TTGTCCACCACGCTGTCAT
*CUBN-F*	TCAGCAAGGATGTGGTGGTT
*CUBN-R*	GTTCCAGCCAAGTTCGCATT
*PLPP3-F*	CTTCGTGTCTGACCTCTTCAAG
*PLPP3-R*	TGTTGTGGTGATTGTTCCTGTC
*MTTP-F*	TGACAGCAGCATTATCCTCCA
*MTTP-R*	CAGCCTTCATTCTGACACAACT
*SLC27A1-F*	ACCACAGGCACCTTCAAGAT
*SLC27A1-R*	CCGAGCAGATGCGAGTGTA
*LTB4R-F*	TCACTATGTCTGCGGAGTCAG
*LTB4R-R*	AGCACAGGCTCATGTTCGT
*KCNN4-F*	ATGCTGCTGCGTCTCTACC
*KCNN4-R*	GCGGAAGCGGACTTGATTG
*SLC7A8-F*	GCCTTCCTGCTGGTCTTCA
*SLC7A8-R*	ATGTCCTCATTAGCCTCCTCTG
*PIK3R2-F*	CACTTGGAAGAGCAGGAGGT
*PIK3R2-R*	AAGCATCTCGGACTAGGAAGG

**Table 2:** Summary of transcriptome sequencing data statistics.

Sample_Name	Raw_Reads	Clean_Reads	Q20 (%)	Q30 (%)
EM1	48,596,854	47,342,498	98.7	95.67
EM2	43,440,298	42,454,230	98.69	95.65
EM3	57,191,982	55,852,078	98.83	96.09
NC1	67,290,316	66,527,492	98.83	96.06
NC2	61,570,630	60,938,366	98.72	95.68
NC3	42,081,762	40,672,606	98.71	95.66

Sequencing yielded > 96% clean reads across all samples, with Q20 > 98.7% and Q30 > 95.6%, indicating high base-calling accuracy. EM3 showed the greatest depth and quality. No quality differences were noted between EM and NC groups.

**Table 3:** Statistics of clean reads alignment rate to the reference genome.

Sample Name	Total Reads	Total Mapped	Multiple Mapped	Uniquely Mapped
EM1	47,342,498	46,430,209 (98.07%)	1,632,481 (3.45%)	44,797,728 (94.62%)
EM2	42,454,230	41,558,282 (97.89%)	1,555,859 (3.66%)	40,002,423 (94.22%)
EM3	55,852,078	54,868,973 (98.24%)	1,797,530 (3.22%)	53,071,443 (95.02%)
NC1	66,527,492	65,366,885 (98.26%)	2,191,223 (3.29%)	63,175,662 (94.96%)
NC2	60,938,366	59,887,762 (98.28%)	2,008,530 (3.3%)	57,879,232 (94.98%)
NC3	40,672,606	39,918,832 (98.15%)	1,277,923 (3.14%)	38,640,909 (95%)

Over 97.8% of clean reads were mapped to the reference genome, with more than 94% uniquely aligned and low multiple mapping rates (3.1–3.7%), reflecting high sequencing accuracy.

**Table 4:** Enrichment of differentially expressed genes in KEGG pathways related to lactation.

Category	KEGG Pathway	DEGs
Amino acid metabolism	Cysteine and methionine metabolism	*PSAT1*, *MPST*, *MTAP*
	Glycine, serine, and threonine metabolism	*GAMT*, *MAOB*, *GRHPR*
	Valine, leucine, and isoleucine degradation	*AACS*, *ACAT2*, *HMGCLL1*
	Arginine and proline metabolism	*PRODH*, *CKB*, *CKMT1B*
Carbohydrate metabolism	Butanoate metabolism	*GAD1*, *OXCT2*, *AACS*
	Pentose phosphate pathway	*TKT*, *RPEL1*
	Galactose metabolism	*AKR1B15*, *AKR1B1*, *GALT*
	Pentose and glucuronate interconversions	*FGGY*, *UGT2A1*, *DHDH*
	Starch and sucrose metabolism	*AMY1B*, *MGAM*, *TREH*, *HKDC1*
Lipid metabolism	Primary bile acid biosynthesis	*CH25H*, *CYP27A1*
	Steroid hormone biosynthesis	*CYP3A7*, *UGT2A1*, *AKR1C3*, *SULT2B1*
	Glycerolipid metabolism	*CEL*, *DGKQ*
Material absorption	Mineral absorption	*TRPV6*, *FXYD2*, *SLC26A6*
	Vitamin digestion and absorption	*PLB1*, *TCN2*, *CUBN*
	Fat digestion and absorption	*CEL*, *SLC27A1*, *PLPP3*, *MTTP*
	Protein digestion and absorption	*SLC7A8*, *KCNN4*, *LTB4R*
	Carbohydrate digestion and absorption	*PIK3R2*, *MGAM*, *HKDC1*, *AMY1B*

**Table 5:** The lactation-related differential metabolites were enriched into the KEGG signaling pathway database.

Pathway ID	Pathway Name	Hits	Compound Name	*p*-Value
hsa04978	Mineral absorption	4	L-Alanine; L-Methionine; L-Asparagine; L-Valine	0.007
hsa04974	Protein digestion and absorption	5	L-Alanine; L-Methionine; L-Asparagine; L-Valine; Tyramine	0.008
hsa00290	Valine, leucine and isoleucine biosynthesis	3	2-Ketobutyric acid; L-Valine; D-erythro-3-Methylmalate	0.023
hsa00270	Cysteine and methionine metabolism	5	L-Alanine; L-Methionine; 2-Ketobutyric acid; S-Adenosylmethioninamine; Methionine sulfoxide	0.034
hsa00970	Aminoacyl-tRNA biosynthesis	4	L-Alanine; L-Methionine; L-Asparagine; L-Valine	0.051
hsa00051	Fructose and mannose metabolism	4	Fructose 1,6-bisphosphate; D-Tagatose 1-phosphate; 2-dehydro-3-deoxy-L-rhamnonate; L-Fucono-1,5-lactone	0.060
hsa02010	ABC transporters	7	L-Alanine; L-Valine; Adenosine; Uridine; Phthalic acid; Xanthosine; Nickel cation	0.081
hsa04152	AMPK signaling pathway	2	Fructose 1,6-bisphosphate; AICAR	0.127
hsa00250	Alanine, aspartate and glutamate metabolism	2	L-Alanine; L-Asparagine	0.175
hsa00750	Vitamin B6 metabolism	2	D-Ribose 5-phosphate; 4-Pyridoxic acid	0.185
hsa00330	Arginine and proline metabolism	3	S-Adenosylmethioninamine; N(omega)-Hydroxyarginine; cis-3-Hydroxy-DL-proline	0.288
hsa04975	Fat digestion and absorption	1	Coenzyme A	0.301
hsa00564	Glycerophospholipid metabolism	2	Citicoline; Demanyl phosphate	0.453
hsa00450	Selenocompound metabolism	1	L-Alanine	0.526
hsa00470	D-Amino acid metabolism	2	L-Alanine; L-Methionine	0.564
hsa00400	Phenylalanine, tyrosine and tryptophan biosynthesis	1	Fructose 1,6-bisphosphate	0.620
hsa00500	Starch and sucrose metabolism	1	3-Ketosucrose	0.641
hsa04977	Vitamin digestion and absorption	1	Coenzyme A	0.660
hsa00340	Histidine metabolism	1	AICAR	0.728
hsa00260	Glycine, serine and threonine metabolism	1	2-Ketobutyric acid	0.735
hsa00360	Phenylalanine metabolism	1	2-Phenylethanol	0.743
hsa00350	Tyrosine metabolism	1	Tyramine	0.886

Differential metabolites related to lactation were enriched in KEGG pathways, mainly involving amino acid metabolism, protein digestion and absorption, and energy-related processes.

## Data Availability

The data presented in this study are available in the main article.

## Abbreviations

The following abbreviations are used in this manuscript:

Abbreviation	Full Term
ABC	ATP-binding cassette (transporters)
AICAR	5-Aminoimidazole-4-carboxamide ribonucleotide
AMPK	AMP-activated protein kinase
ATF4	Activating transcription factor 4
CCK-8	Cell Counting Kit-8
CSN1S1/CSN2/CSN3	Casein genes (alpha-S1, beta, and kappa-casein)
DEG	Differentially expressed gene
DMEM/F-12	Dulbecco’s Modified Eagle Medium/Nutrient Mixture F-12
EdU	5-ethynyl-2′-deoxyuridine
EIF2AK1/EIF2AK4	Eukaryotic translation initiation factor 2 alpha kinase 1/4
EM	Methionine-containing dipeptide
FBS	Fetal bovine serum
GO	Gene ontology
KEGG	Kyoto Encyclopedia of Genes and Genomes
LC-MS/MS	Liquid chromatography–tandem mass spectrometry
MCF-10A	Human mammary epithelial cell line MCF-10A
mTOR	Mammalian target of rapamycin
PBS	Phosphate-buffered saline
PCA	Principal component analysis
PepT1	Peptide transporter 1
PERK	Protein kinase RNA-like endoplasmic reticulum kinase
PI3K	Phosphoinositide 3-kinase
PLS-DA	Partial least squares discriminant analysis
qRT-PCR	Quantitative real-time polymerase chain reaction
RNA-seq	RNA sequencing
SAM	S-adenosylmethionine
VIP	Variable importance in projection
